# An in vitro protocol for rapidly assessing the effects of antimicrobial compounds on the unculturable bacterial plant pathogen, *Candidatus* Liberibacter asiaticus

**DOI:** 10.1186/s13007-019-0465-1

**Published:** 2019-07-31

**Authors:** Joseph Krystel, Qingchun Shi, Jefferson Shaw, Goutam Gupta, David Hall, Ed Stover

**Affiliations:** 1 Subtropical Insect and Horticulture Research Unit, US Horticultural Research Laboratory, 2001 S. Rock Rd, Ft. Pierce, FL 34945 USA; 2 New Mexico Consortium, 100 Entrada Dr, Los Alamos, NM USA

**Keywords:** Unculturable bacteria, Bioassay, Anti-microbial peptide, *Candidatus* Liberibacter asiaticus, Huanglongbing, Citrus, Asian citrus psyllid

## Abstract

**Background:**

Most bacteria are not culturable, but can be identified through molecular methods such as metagenomics studies. Due to specific metabolic requirements and symbiotic relationships, these bacteria cannot survive on typical laboratory media. Many economically and medically important bacteria are unculturable; including phloem-limited plant pathogens like *Candidatus* Liberibacter asiaticus (CLas). CLas is the most impactful pathogen on citrus production, is vectored by the Asian citrus psyllid (ACP, *Diaphorina citri*), and lacks an effective treatment or resistant cultivars. Research into CLas pathogenicity and therapy has been hindered by the lack of persistent pure cultures. Work to date has been mostly limited to *in planta* studies that are time and resource intensive.

**Results:**

We developed and optimized an in vitro protocol to quickly test the effectiveness of potential therapeutic agents against CLas. The assay uses intact bacterial cells contained in homogenized tissue from CLas-infected ACP and a propidium monoazide (PMA) assay to measure antimicrobial activity. The applicability of PMA was evaluated; with the ability to differentiate between intact and disrupted CLas cells confirmed using multiple bactericidal treatments. We identified light activation conditions to prevent PCR interference and identified a suitable positive control for nearly complete CLas disruption (0.1% Triton-X 100). Isolation buffer components were optimized with 72 mM salt mixture, 1 mM phosphate buffer and 1% glycerol serving to minimize unwanted interactions with treatment and PMA chemistries and to maximize recovery of intact CLas cells. The mature protocol was used to compare a panel of peptides already under study for potential CLas targeting bactericidal activity and identify which were most effective.

**Conclusion:**

This psyllid homogenate assay allows for a quick assessment of potential CLas-disrupting peptides. Comparison within a uniform isolate largely eliminates experimental error arising from variation in CLas titer between and within individual host organisms. Use of an intact vs. disrupted assay permits direct assessment of potential therapeutic compounds without generating pure cultures or conducting extensive *in planta* or field studies.

**Electronic supplementary material:**

The online version of this article (10.1186/s13007-019-0465-1) contains supplementary material, which is available to authorized users.

## Background

The vast majority of bacteria have not or cannot be cultured through traditional means [1, 2]. As a result, the overwhelming majority of the predicted number of microbes have escaped meaningful description; only about 12,000 of the 10^7^ to 10^9^ predicted species having been so described [3, 4]. Uncultured and uncharacterized species include bacteria with a high potential importance; such as bacteria found in the human gut [5], bacteria associated with water [6], and bacteria in soil or symbiosis with agricultural crops [7–9]. Most methods to investigate pathogenicity are based on the use of pure cultures in high concentration, and are therefore unsuitable for unculturable pathogens. Completion of Koch’s postulates to unequivocally verify the causal agent for diseases depends on an isolated and pure culture [10, 11].

Bacterial communities often display obligate interspecies interactions. In communities, many metabolites and gene products are released into the environment where they can be absorbed and utilized by other species. These interactions favor species with streamlined genomes that omit the cellular machinery to produce already available resources [12]. The more supportive and stable the local environment, the more likely a bacterial species is to have lost otherwise necessary genes. Bacteria that are endosymbiotic or exist primarily in biofilms have thus undergone loss of many functions that would be essential for independently viable organisms, compromising ability to be cultured [13, 14]. Advances are being made to grow such microbes through use of genomic data to tailor media to the microbes’ metabolic needs [15]. This analysis has begun in CLas with the report of a full genome sequence and analysis to identify deficient metabolic pathways [16, 17]. Other efforts with mutually dependent strains, that are not individually amenable to pure cultures, include being grown through co-culture or the maintenance of communities in biofilms [18, 19].

Research into unculturable pathogenic bacteria require alternative means of identifying viable and living microbes. The common description used to quantify populations of a microbe is the number of colony forming units (CFUs); where a single bacterium is considered alive and viable if it forms a distinct colony of cells when placed on a suitable culture material at appropriate dilution. For bacterial species that cannot be cultured, this is clearly not suitable, requiring less direct means to characterize populations. Among the methods that have been used are assessments of: deoxyribonucleic acid (DNA) transcription/translation activity, metabolic activity, and maintenance of intact cell membranes [20].

The assessment of cell membrane integrity has been conducted through a variety of means, mostly involving preferential exclusion or uptake of dyes or markers. Recently described techniques combine selective exclusion of DNA-binding dyes and quantitative polymerase chain reactions (qPCR). A dye such as ethidium monoazide (EMA), propidium monoazide (PMA) or a derivative is added to a sample where they strongly bind DNA. When activated through light excitation, the dyes form a covalent crosslink with the DNA and render it unavailable as PCR template. Because the dyes do not penetrate cell membranes, any DNA within an intact cell is unaffected. Only free DNA in solution, or in cells with compromised membranes, will be bound by the treatment. Following this procedure, a viability q-PCR (v-qPCR, as the intact cells are putatively viable), can measure the number of intact cells through amplification of DNA targets [21, 22]. One disadvantage of v-qPCR is the possible underestimation of cell mortality, if it is not associated with cell membrane disruption [20]. Of the commonly available v-PCR dyes, PMA is reported to be more likely to underestimate cell disruption, suggesting results be considered the maximum potential number of viable cells and therefore protocols should focus on maximizing the suppression of signal from dead cells [20, 23]. PMA’s ability to penetrate non-viable but largely intact cells may be complicated by the presence of a large number of dead cells lowering the availability of PMA molecules to each cell. In previous reports, these problems were mostly absent or ameliorated in experiments using combinations of appropriate wavelength light sources, sufficient incubation time, PMA concentration, light exposure, and a ratio of dead to living cells of less than 10,000:1 [23, 24].

Psyllids and leafhoppers are by far the most important vectors of bacterial pathogens to plants [25]. The bacterial pathogens they transmit, like *Candidatus* Phytoplasma spp. (phytoplasmas) [26] and *Candidatus* Liberibacter spp. [27] represent a major threat to food crops worldwide. These phloem limited pathogens; both the cell-wall lacking phytoplasmas and the gram negative proteobacteria (Liberibacters) have historically proven very resistant to laboratory culturing [13]. The *Candidatus* status indicates characterization primarily through genetic means and without culturing [28]. *Candidatus* Liberibacter asiaticus (CLas), a fastidious member of the Liberibacters, is now widely accepted as the associated agent of huanglongbing (citrus greening, HLB). HLB represents the most pressing and dangerous disease in citrus agriculture. Although first described ~100 years ago in Asia, recent developments have led to a tremendous outpouring of research on combatting this disease. An increase in global transportation has spread the pathogen and its associated insect vector; where in some citrus growing regions they have had a devastating effect. There is currently no effective treatment for HLB and no commercial cultivar with meaningful resistance, but efforts are underway to develop methods to test and deploy both [27, 29]. The verification of CLas as the HLB pathogen and subsequent studies of the disease have been complicated by its unculturable nature. CLas titers in plant and insect hosts are variable and somewhat unpredictable, hindering in vivo studies where uniform inoculation and infection is needed. When feeding on infected plant material, ACP have shown a high rate of infection with CLas but greatly varied titers [30]. A consistent source and alternative testing method is needed for CLas and similar pathogens. Here we describe an in vitro assay on bacteria in an insect-host homogenate to provide a uniform sample for treatment and controls. It is a cost effective and time efficient protocol for testing therapeutic compounds acting through cell lysis or membrane disruption.

## Methods

**Fig. 1:**
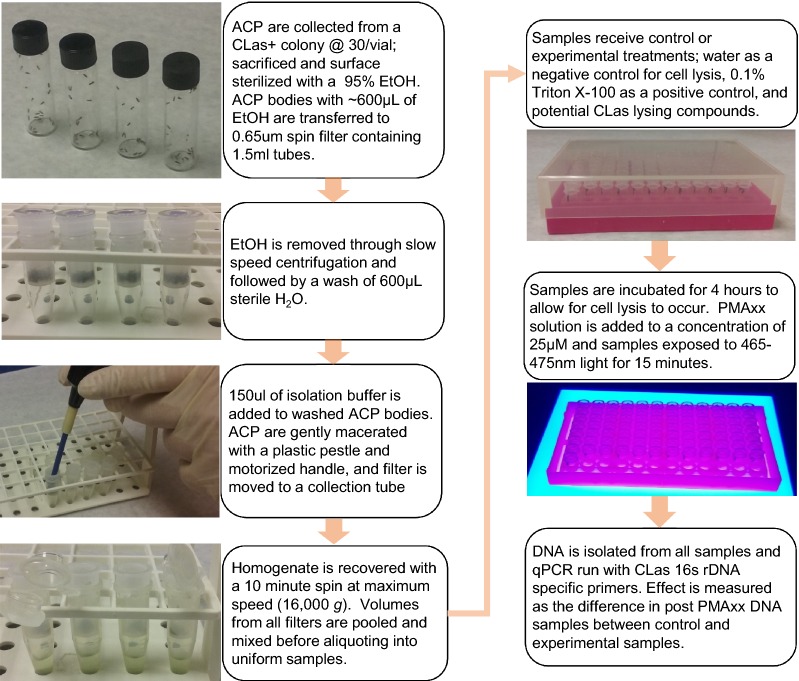
Procedure for antimicrobial peptide assay on *Candidatus* Liberibacter asiaticus with insect homogenate

All experiments were conducted at least twice with consistent results unless otherwise described. An overview of the finalized protocol is shown in Fig. 1. A printable protocol is provided in Additional file 1.

### Psyllid collection

The ACP were collected from colonies maintained at the United States Department of Agriculture (USDA) U. S. Horticultural Research Laboratory in Fort Pierce, Florida. The ACP were raised and fed on CLas + citrus plants with CLas levels checked on a bi-weekly basis. Thirty mature adult psyllids were collected by suction into glass vials for CLas isolation.

### Isolation of intact CLas cells from ACP homogenate

The following extraction protocol was based on the successful isolation of *Wolbachia* spp. from adult drosophila [31] with many modifications. Each isolation yields approximately 150 µl of CLas containing homogenate. For experimental designs requiring a larger volume of homogenate, multiple isolations of 30 ACP collected at the same time, were pooled to generate a uniform mixture.

ACP were stored at 4 °C for several hours to anesthetize them and facilitate handling. The psyllids were briefly submerged in a 95% ethyl alcohol (EtOH) solution for surface sterilization and euthanasia. Psyllid bodies were transferred through pipetting into a 0.65 µm spin column with approximately 600 µl of EtOH. Tubes were spun on a tabletop micro-centrifuge (5 s @ 1000 g) to remove the majority of the EtOH solution. Next, 600 µl of sterile water was passed through the filter to remove any remaining EtOH (5 s @ 1000 g). Filters were moved to a new sterile collection tube where 150 µl of isolation buffer (21 mM KCl, 36 mM NaCl, 15 mM MgSO_4_, a 1 mM phosphate buffer @ pH 7 (K_2_HPO_4_ + KH_2_PO_4_) and 1% glycerol) was added. The ACP were gently macerated using a pellet pestle and motorized hand-held driver until no intact ACP bodies were visible. CLas containing homogenate was recovered through centrifugation for 10 min @ 16,000*g*. The filter was discarded and the flow-through mixed through gentle vortexing. A 5 µl sample of this suspension was reserved for initial CLas titer assessment and the remaining volume subjected to peptide or control treatments. The homogenate was divided into aliquots for negative control, positive control, and each peptide tested. Sterile nuclease free water was used as the negative control for cell lytic activity and polyethylene glycol tert-octylphenyl ether (Triton-X 100) at 0.1% as the positive control. After treatments were added to initial aliquots, the cell suspension was further divided into individual samples of 5 µl contained in either each well of a 96 well plate or each tube in 8-tube strips of 0.2 ml microcentrifuge tubes, depending on number of treatments compared.

### PMAxx treatment and v-qPCR

Samples were incubated for 4 h before viability assay with PMAxx (Biotium). PMAxx is a proprietary modified propidium monoazide dye designed to more effectively bind free DNA and improve the discrimination between putatively live and dead cells [32, 33]. Each 5 µl sample was supplemented with 1 µl of 0.15 mM PMAxx, to bring the final concentration to the manufacturer recommended 25 µM concentration. The no PMAxx controls instead received 1 µl water. All samples were mixed through tap-spin and allowed to incubate at room temperature for 5 min. They were exposed on the Glo-Plate blue LED illuminator (Biotium) for 15 min to crosslink free DNA in either a transparent 96 well plate or in 0.2 ml strip tubes placed in an open bottom rack.

Immediately following PMAxx treatment, DNA was isolated from all samples. For the extraction, 45 µl of lysis buffer (Tris–EDTA, 0.1% SDS, 0.05% Tween-20) was added to each sample, followed by 50 µl of phenol buffered to pH 7–8. Samples were mixed through vortexing, spun at 16,000*g* for 10 min and then the aqueous phase transferred to a new 96 well plate or 8-tube strip. DNA was precipitated with half volume of 7.5 M ammonium acetate and three volumes of ice cold 95% EtOH. Samples were gently mixed through inversion and incubated at 25 °C for 10 min. The DNA was pelleted with a 10 min centrifugation at 16,000*g* in a tabletop centrifuge. The supernatant was decanted and the pellet air dried for ~ 15 min before being dissolved in 50 µl of nuclease free water. A 1 µl volume of DNA solution was taken for qPCR analysis.

Cycle threshold (C_t_) values were obtained through qPCR analysis. The qPCR was conducted with Taqman fast chemistry with multiplex capable primers targeting the CLas 16 s ribosomal DNA (rDNA) with the sequences; 5′-AGGCTCTTACCCTACCAACTA-3′, 5′-GCATGGAAACGTGTGCTAATAC-3′ and the probe 5′-56-FAM/TTGGAGAGA/ZEN/GATGAGCCTGCGTTG/3IABkFQ/-3′. This primer and probe set has a calculated standard curve equation of C_t_ = − 3.282Log(Copy#) + 38.078, r^2^ = 0.997 and an efficiency of 101.7% as conducted on an 7500 Real-Time PCR System (Applied Biosystems).

### Statistical analysis

Statistical analysis was conducted in the JMP Genomics suite from the SAS Institute. Data was compared by the Wilcoxon non-parametric analysis with a significance threshold of p < 0.05. If a v-qPCR reaction resulted in an undetermined C_t_, the maximum PCR cycle number of 40 was provisionally substituted and Quantile analysis run. If this provisional value was above the upper fence threshold (Upper quartile plus one and a half times the interquartile range, Q3 + 1.5IQR), it was not included in mean calculations.

## Results

### Development of the in vitro assay using ACP homogenate

**Fig. 2:**
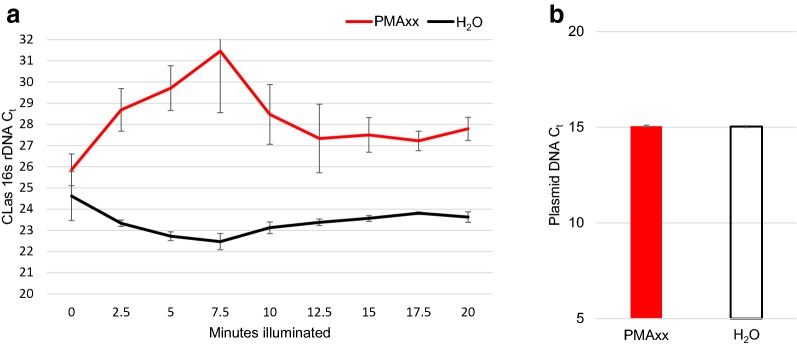
Time trial for full activation of PMAxx with LED illuminator and PMAxx concentration test. **a** CLas containing ACP homogenate was aliquoted and treated either with 25 μM PMAxx or water and illuminated for 0, 2.5, 5, 7.5, 10, 12.5, 15, 17.5 or 20 min. Samples were immediately taken for DNA extraction after treatment and taken as template for qPCR analysis of CLas 16 s rDNA. Three replicates for each treatment at each time point were run with the values averaged and shown with standard error of the means. The difference between PMAxx treated and untreated samples levels off after 12.5 min. **b** A group of homogenate aliquots was subjected to the final protocol PMAxx treatment conditions (25 μM PMAxx or water and 15 min illumination). All samples were supplemented with 20 pg of plasmid DNA after PMAxx treatment and qPCR amplified with plasmid specific primers. Shown are the average C_t_ values from 7 replicates with standard error of the means. Plasmid amplification as measured by C_t_ was not statistically different in the presence of PMAxx treated samples compared to water, indicating no effect of residual PMAxx

Peak excitement of PMAxx occurs at ~ 464 nm and is inactivated through crosslinking to bound DNA or reaction to water. If PMAxx dye remains active in the sample after light treatment, it may interfere with the PCR by binding DNA released in the DNA extraction protocol or by interfering with template amplification by DNA polymerase [33, 34]. Full reaction of free PMAxx prior to PCR is therefore necessary for accurate v-qPCR analysis. In order to ensure complete inactivation, we performed a time trial to identify optimal time conditions. Homogenate from two sets of 30 ACP were pooled and aliquoted. Replicated samples were treated with 25 µM PMAxx or water and illuminated for durations between 0 and 20 min at an interval of 2.5 min. Light treatment was carried out at room temperature on the Glo-Plate Blue with blue LEDs generating light between 465 and 475 nm, an approximate range to the optimal wavelength. All samples then underwent DNA extraction and qPCR analysis (results in Fig. 2). Samples exposed to light for 2.5 to 10 min showed a relative reduction in intact cell signal with (ΔC_t_ of 5–9 for PMAxx treatment) compared to those exposed longer. Samples illuminated for 12.5 to 20 min returned a consistent ΔC_t_ between PMAxx treatment and controls. The closely related *Liberibacter crescens* (Lcr) has a reported doubling time of 36.7 h @ 28 °C [35], so a true increase in cell number over only 4 h was deemed unlikely. We therefore concluded that the lower signal of live cells from 2.5 to 10 min of light exposure represented interactions between unreacted PMAxx and the qPCR chemistry. The PCR disruption from the lower duration of light exposure was not present in all replicates, but the uniformity in v-qPCR at all time points 12.5 min or longer was consistent. Based on these results, we selected 15 min as an appropriate time point that reliably did not interfere with PCR efficiency.

A PMAxx concentration of 25 µM is the manufacturer suggested working concentration. However, higher concentrations of PMA have been reported to inhibit PCR reactions independent of light exposure [36]. An additional experiment was conducted to determine if any lingering components from the PMAxx treatment under our experimental conditions would inhibit downstream qPCR. Replicated samples of homogenate were treated with either 25 µM PMAxx or water control and exposed to light for 15 min. Samples were then supplemented with 20 pg of purified plasmid DNA as a qPCR template. A qPCR analysis with plasmid specific primers was conducted on all samples. No statistical difference in C_t_ values was seen between PMAxx and non-PMAxx extractions, indicating the given conditions (25 µm PMAxx, 15 min light, and presence of ACP homogenate matrix) did not reduce qPCR efficiency on intact template (Fig. 2).

**Fig. 3:**
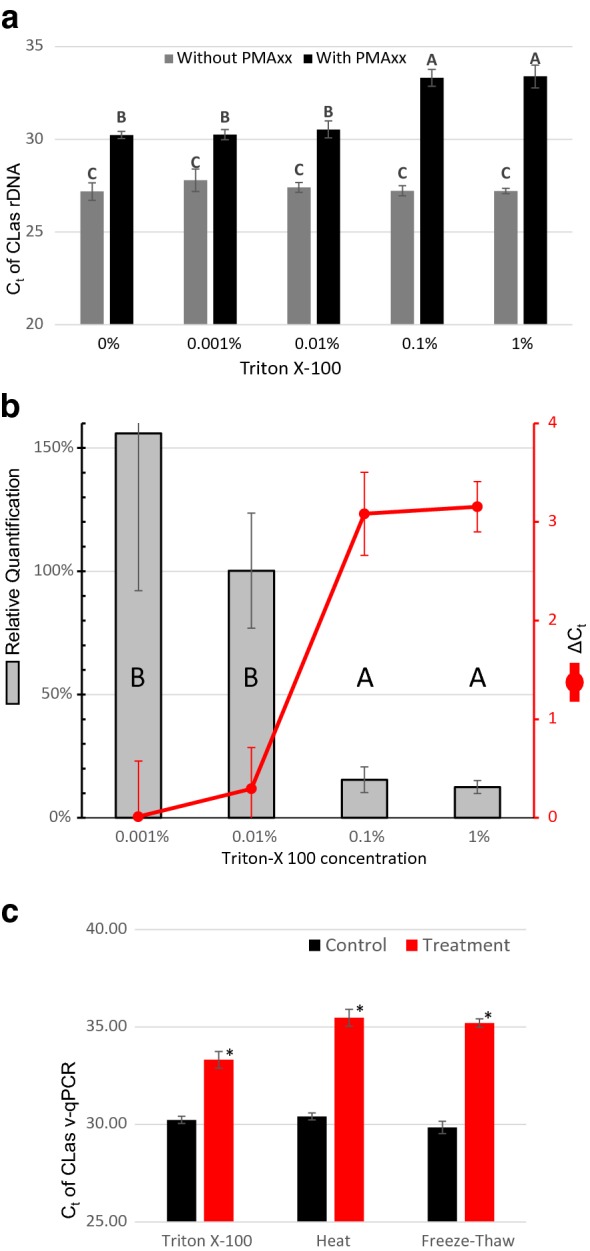
Dose response curve to Triton-X 100 positive control and comparison to other cell lysis methods. **a**, **b** CLas containing ACP homogenate was treated with a serial dilution of the detergent Triton-X 100 (1–0.001%) or a control of sterile water (0%) for 4 h. C_t_ values for v-qPCR from each combination of treatments shown in **a** as the average of six replicates. Relative quantification of cells under each treatment compared to the untreated control and ΔC_t_ in PMAxx averages is shown in **b**. CLas cells in homogenate were also heat stressed for 15 min @ 95 °C or subjected to three rounds of freezing on liquid nitrogen followed by thawing in a 37 °C water bath. **c** is a comparison between the v-qPCR C_t_ values for each treatment compared to 4 h of 0.1% Triton-X 100. Each treatment is paired with a non-treatment control; Triton-X 100 with water and each temperature stress to an identical time period @ 25 °C. All three cell lysis treatments (marked with “*”) were statistically different than the paired control (P < 0.05)

We selected the non-ionic detergent Triton-X 100 as a positive control for cell disruption. A dose response experiment was conducted to identify an appropriate concentration for lysing CLas cells. A CLas containing homogenate was divided into five groups of twelve samples. Groups were treated with a serial dilution of Triton-X 100 from 1% down to 0.001% v/v or a water control. After a 4 h incubation, samples underwent v-qPCR analysis. There was no significant difference in qPCR quantification of total CLas DNA between all treatments when no PMAxx was used. Intact CLas as shown with PMAxx and v-qPCR in 0.001% and 0.01% Triton-X treatments, was statistically unchanged from a water only treatment. Treatment with either 0.1% or 1% Triton-X resulted in a significant decrease in intact cells (higher C_t_) compared to both the water and the lower Triton-X concentration treatments (Fig. 3). The average ΔC_t_ for the 0.1% and 1% Triton-X treatments compared to control was 3.12, representing an approximately 90% reduction for intact cells (Fig. 3). We selected 0.1% as the appropriate concentration for a positive control of cell lysis. This concentration showed the same efficacy as 1% while reducing potential interactions with PMAxx, qPCR or trial peptide chemistries.

The effectiveness of these v-qPCR conditions on CLas cells in ACP homogenate was confirmed on cells lysed through heat and freeze/thaw treatments. Replicated samples were treated with either heat (15 min @ 95 °C) or 3 cycles of freeze/thaw (alternating liquid nitrogen and 37 °C water bath) with control samples maintained at 25 °C. Both temperature stress treatments resulted in a statistically significant decrease for intact cell signal in v-qPCR of > 5C_t_ (Fig. 3). Because of the differing time requirements, mode of action, and reported lower correlation to cell viability [37], temperature stress was eliminated as a positive control for this homogenate assay.

**Fig. 4:**
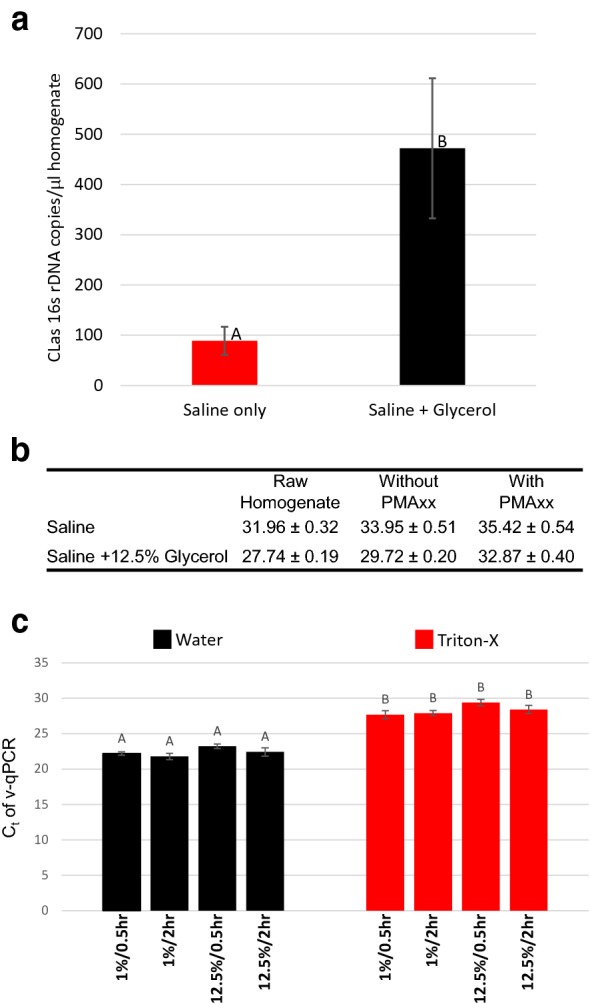
Effects of glycerol buffer concentration on CLas viability. **a**, **b** Homogenate was prepared with saline buffer or saline buffer + 12.5% glycerol. Relative quantification of intact cells by v-qPCR from two trials shown in **a** with the corresponding average qPCR values of raw homogenate, light without PMAxx and light with PMAxx in **b**. The average viable cell signal from saline + glycerol was statistically higher than saline only in non-parametric analysis when comparing six biological replicates. **c** Viability of CLas cells before and after Triton-X 100 treatment was tested in homogenates with 1% and 12.5% glycerol. Values and statistical groupings (P = 0.05 in non-parametric analysis). There was no statistical change on total cell isolation (No PMAxx control), viable cell (Water/PMAxx) or lysis through detergent (Triton-X/PMAxx) between glycerol concentrations. Values shown are the average of five biological replicates

The addition of glycerol to the isolation buffer was explored to increase the quantity of intact CLas cells recovered. Efforts from a collaborator on isolating CLas indicated that 12.5% glycerol was more effective at recovering intact cells when compared to phosphate buffered saline (PBS) as determined through electron microscopy (Michelle Heck, unpublished). An isolation was conducted using buffers with and without 12.5% glycerol. The glycerol containing buffer yielded an isolate with a statistically significant increase for intact cells (Fig. 4). A statistically higher average C_t_ in raw homogenate and no-PMAxx controls was also seen in multiple repeated experiments.

Glycerol concentrations of 1% and 12.5% were next compared for their effects on intact cell isolation and any interference with Triton-X 100 activity (Fig. 4). In samples collected 0.5 h or 2 h after isolation and treatment, there was no statistical difference between v-qPCR on intact cell isolation (water added as a control) or the ability to cause cell lysis through treatment (Triton-X 100 0.1%). Because 1% glycerol yielded isolates with the same quantity of intact CLas cells and did not change the ΔC_t_ seen with Triton-X 100 treatments, it was selected for the final buffer composition.

### Application of homogenate assay for candidate peptides

**Fig. 5:**
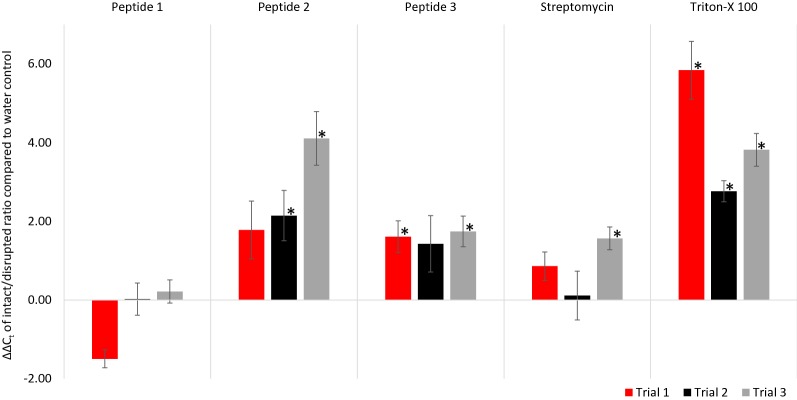
Testing of synthetic antimicrobial peptides on CLas. Summary data from assessment of three putative AMPs, 1 mM streptomycin and 0.1% Triton-X 100. All samples underwent a 4 h incubation and received PMAxx @ 25 μM with light @15 min. The results from three separate experiments are summarized here. Each experiment contained twelve replicate samples per treatment and control. Values (ΔΔC_t_) are shown as the average difference between the intact/disrupted cell ratio (ΔC_t_ of CLas 16 s rDNA from PMAxx compared to no PMAxx) for the experimental treatment compared to the intact/disrupted cell ratio of the no peptide (water) control. * Results with statistically significant increase in ΔΔC_t_ compared to no-peptide control (P < 0.05) by non-parametric comparison

The optimized homogenate assay was used to test a selection of putative anti-microbial peptides (AMP) already being researched in the lab for killing of CLas and treatment of HLB (Fig. 5). Peptide 1 and peptide 2 are citrus-derived anti-microbial short peptides with modifications based on biophysical modeling to improve disruption of gram negative bacterial cell membranes and reduce susceptibility to protease degradation. Peptide 2 has previously shown promising results in detached tissue assays on CLas infected citrus tissue and in in vitro assays for inhibition of growth against *E. coli* and *Xanthomonas* spp. Peptide 3 is an insect derived peptide that has also shown antimicrobial activity. Additional details of these peptides cannot be reported further until patent applications are complete. Also included was streptomycin, a widely used antibiotic that is applied in Florida for controlling CLas [38], as well as the positive and negative controls as described above. CLas cells were collected in the pooled homogenates of 120 ACP filtered in groups of 30. Peptide treatments and streptomycin were run with 12 replicated samples each maintained at 1 mM concentrations for 4 h @ 25 °C. Three independent assay experiments were run with the same experimental design.

For each treatment (control or experimental peptide), an intact/disrupted cell ratio was calculated as difference between C_t_ values of the PMAxx and no-PMAxx samples (ΔC_t_). Treatment effects were then assessed as the relative change in the intact/disrupted cell ratio as compared to the water negative control (ΔΔC_t_). Of the three experimental peptides tested; peptide 2 and peptide 3 showed a statistically significant increase in CLas disruption (greater ΔΔC_t_) and were selected for progressing into *in planta* studies. Peptide 1 treatment resulted in no increase in CLas disruption (ΔΔC_t_ ≈ 0) and was dropped from consideration as an HLB therapeutic. Streptomycin treatment yielded some increase in ΔΔC_t_, but was not consistent between trials. Any effect from streptomycin was expected to be modest as it targets ribosomal subunits rather than directly lysing cells [39]. Treatments acting without directly destabilizing cell membranes tend to result in overestimation of viable cells or underestimate the rate at which cells are killed when using v-qPCR ([21]. The Triton positive controls consistently resulted in a statistically significant increase in cell disruption (greater ΔΔC_t_) over the course of the trials.

## Discussion

This psyllid homogenate assay now plays an important role in our pipeline for testing CLas-killing peptides, for either exogenous application or delivery as transgenes. Work on whole trees requires a much longer experimental run, as citrus are slow-growing woody plants that take many months to develop HLB symptoms. Using individual plants increases the variability between replicates due to the complex nature of the pathogen/host interactions. Whole tree experiments also have substantial costs since each tree needs many work-hours for maintenance, higher amounts of therapeutic materials to attain adequate phloem concentrations, and are vulnerable to storm and cold damage. While intact plant trials will remain essential for determining therapeutic effect, treatment concentrations and phytotoxicity, the time and costs for such trials are so great that early elimination of less successful CLas-killers is invaluable.

We selected PMAxx for this assay to minimize false positive results in on our experimental platform. PMA dyes have been found to be less influenced than EMA by variation in bacterial cell membranes between species. This is advantageous for an assay which may be applied to a range of poorly characterized unculturable microbes. PMA is less efficient at suppressing the PCR signal of compromised cells then EMA, but also less likely to penetrate a viable cell [23]. PMA treatments are generally considered to be a conservative measurement of killed cells with a much higher tendency to overestimate living cell numbers [23]. Therefore, PCR results after PMA treatment should be considered as reporting a maximum value for the remaining viable/intact cells with the actual number possibly being smaller. This is preferred in this assay because downstream validation of CLas-killing therapies will require costly and time intensive *in planta* or field studies, and we would rather underestimate than overestimate treatment effects.

Conditions reported for using PMA and other photo-reactive viability dyes are widely varied. Many factors can influence the efficiency of the reaction and the magnitude of the signal generated. These factors have been extensively reviewed by Fittipaldi et al. [23]. Some conditions can be easily adjusted in developing a procedure for an individual bacterium; such as light wavelength, exposure time, PMAxx or other dye concentration, and incubation times. Some conditions are specific for individual assay systems and cannot easily be changed; like the membrane structure of the bacteria tested and the matrix in which the bacteria are collected. Here we have identified a set of lighting conditions and buffer composition suitable for testing therapeutic compounds against intact CLas cells in an ACP-homogenate matrix.

The isolation is conducted in a dilute phosphate buffered saline solution containing glycerol. Trials with complex culture media components (data not shown) proved inconsistent, so a minimal saline solution was selected to reduce potential interactions between peptide treatment and buffer components. A pH buffering capacity was included to avoid pH effects as a complicating factor in maintaining intact CLas, influence on peptide action, and PMAxx function. Tests were conducted to identify the minimum concentration of phosphate salts needed to maintain a stable pH while avoiding osmotic stress on the CLas cells. A concentration of 1 mM was found to effectively buffer pH in both positive and negative controls without impacting intact cell numbers (data not shown). We also tested the inclusion of glycerol in the buffer composition. Glycerol is a well-known cryoprotector, anti-oxidant, and protein stabilizer. It has been identified as being highly produced in cells subjected to temperature and water stress [40] and the inclusion of glycerol in buffers is reported to improve the viability and metabolic activity of bacterial cells [41]. Several trials with early buffer compositions showed a larger PMAxx effect (greater cell disruption) at early time points that lessened if cells were assayed after a recovery period. We theorized that this discrepancy was due to a transient disruption of cell membranes through mechanical or osmotic stress allowing PMAxx to penetrate otherwise intact cells. This was not seen in trials with the final buffer composition containing glycerol. Adding glycerol also resulted in a significant increase in the recovery of intact CLas cells across all experiments and an increase in total DNA recovered in some experiments. The mode of action of the glycerol is not clear. We postulate that it may increase intact cell numbers by protecting cell membranes from stress during isolation. It is also possible that glycerol improves cell recovery during transfer steps by altering homogenate viscosity. This is supported by the decreased average C_t_ (higher cell numbers) in raw homogenate and no-PMAxx controls when glycerol is included (Fig. 4b). Further research will be required for the mode of action to be conclusively determined.

In this study, the use of 0.1% Triton-X 100 as a positive control offered an ideal range of effects. It generated a consistent and statistically significant lysis effect between 85 and 98% (ΔΔC_t_ 2.8–5.8) compared to water controls. It is unlikely that such a treatment would disrupt 100% of cells as some CLas cells are likely associated with resistant biofilms. Stronger detergents such as SDS may have generated a higher ΔΔC_t_, but generating 100% cell lysis would result in undetermined C_t_ values through v-qPCR analysis. Such a result may be incorporated at the theoretically lowest possible concentration detectable (C_t_ 40 for 40 cycle reactions). However, if all control replicates resulted in undetermined values, it would be difficult to rule out the possibility of failed amplification from experimental error. Triton-X 100 acts through cell membrane disruption and has a similar mode of action as predicted for some of the assayed peptides. Published studies on PMA have found that detergent based agents provide the highest correlation between v-qPCR results and culturability, when it is possible to test both in parallel [37]. This is likely because the detergents directly impact permeability of the membrane to the dye. Other methods, such as antibiotic treatments, may result in a non-viable cell that still possesses membrane intact enough to exclude it. This cannot be experimentally replicated with CLas without a culturing protocol. However, we judged that detergents were most likely to provide the best relationship between cells successfully disrupted by the positive control and viability.

All three of the peptides tested as potential CLas-killers were initially selected by the preliminary analysis methods available (in silico interaction, biophysical modeling, and tests against culturable gram negative bacteria). Under currently available protocols, it would not be possible to further differentiate between them without *in planta* studies. The homogenate assay was able to directly measure the impact of the three proposed therapeutic compounds on the intact/disrupted ratio of CLas cells. The effectiveness of each treatment was compared to a positive control acting through analogous activity and in parallel to a commercial treatment. Peptide 2 and peptide 3 treatments were shown to significantly reduce intact CLas cells in a method and timeframe similar to culture based assays available for other bacteria. Initially analysis was conducted by comparing the post PMAxx C_t_ values of treatment and control samples. However, in subsequent use of the protocol, it was found that some peptide treatments resulted in higher or lower average DNA yields in both the PMAxx and no-PMAxx samples (data not shown). To account for any changes in extraction or qPCR efficiency caused by experimental treatments, the analysis was changed to use the ΔΔC_t_ method described in the results.

Adult psyllids were used as a reliable and practical source of intact CLas cells. We also tested the suitability of collecting cells from the tissue of 4th and 5th instar ACP nymphs. While late stage nymphs have been reported to support high titers of CLas cells [30], this protocol yielded very low initial concentrations when performed on a comparable mass of nymphal tissue (data not shown). This may be due to bacteria residing in different tissues of nymphs that results in loss of CLas in the insect debris, or greater sensitivity to disruption for CLas in the nymphal matrix. A more detailed study using nymphs of uniform ages and development would be informative, but the use of adults maximizes the ease of selection and handling while maintaining a high bacterial concentration.

The total number of adult ACP used in each assessment was based on the experimental design and influenced by the condition of the insect colony. This protocol requires sufficient volume and CLas concentration of homogenate for each treatment. When more replicates or treatments were desired, additional groups of ACP were homogenized and pooled. A typical run included 4 tubes of psyllids recovering 600 µl of total homogenate. This volume is sufficient for simultaneous testing of four peptides with both positive and negative controls. If the initial C_t_ value or routine psyllid colony assessment indicates a lower titer of CLas, additional tubes with less isolation buffer can be used to increase the concentration of intact bacteria. Using higher numbers of ACP per tube was attempted, but the additional tissue obstructed the filter and resulted in an inability to recover homogenate.

## Conclusions

Research on HLB has been limited by the availability of bacterial cell cultures for experimentation. Work has focused primarily on *in planta* experiments with either adult trees or detached tissue. However, these experiments have many limitations that increase the costs and time requirements. The v-qPCR based assay described here offers distinct advantages for testing potential CLas-killing compounds in a rapid workflow at reasonable material costs and research effort. This allows a larger number of treatments to be tested with more replicates. A trial can be setup, run, and analyzed in a single day, compared to weeks or years for detached tissue and greenhouse studies. The small volume of each replicate requires a lower quantity of potential therapeutics; many of which are novel compounds with expensive synthesis or extraction protocols. This permits the screening of a wider range of chemicals for CLas lysing activity to determine which compounds are suitable for more detailed investigation. Replicating this initial selection for all potential therapeutics *in planta* would be prohibitively expensive and require additional time—while HLB continues to devastate the citrus industry.

## Additional file

**Additional file 1.** Protocol of Clas isolation, incubation and PMAxx treatment, DNA extraction.

## Data Availability

The datasets used and analyzed during this study are available from the corresponding author on reasonable request.

## Abbreviations

ACP: Asian citrus psyllid
CFU: colony forming units
CLas: *Candidatus* Liberibacter asiaticus
C_t_: cycle threshold
DNA: deoxyribonucleic acid
EMA: ethidium monoazide
EtOH: ethyl alcohol
HLB: huanglongbing
Lcr: *Liberibacter crescens*
PBS: phosphate buffered saline
PCR: polymerase chain reactions
PMA: propidium monoazide
qPCR: quantitative polymerase chain reactions
rDNA: ribosomal DNA
USDA: United States Department of Agriculture
v-qPCR: viability q-PCR
