# Supplementary material for: An in vitro protocol for rapidly assessing the effects of antimicrobial compounds on the unculturable bacterial plant pathogen, Candidatus Liberibacter asiaticus
Source: Plant Methods. 2019 Jul 31;15:85. doi: 10.1186/s13007-019-0465-1 (PMC6668101; doi:10.1186/s13007-019-0465-1)
Supplement: Supplementary file 1 — Additional file 1. Protocol of Clas isolation, incubation and PMAxx treatment, DNA extraction. [file 13007_2019_465_MOESM1_ESM.pdf]

Protocol:

Clas isolation:

1. Collect 30 CLas+ psyllid per tube and drown them in 600uL of 95%EtOH
2. Transfer psyllid bodies to Centrifugal Filters (0.65 micrometer spin column).
3. Spin for 5-10 seconds in tabletop centrifuge at low speed setting (around 1,000 g), discard flow through.
4. Add 600uL sterile H_2O to the filter, spin 5-10 seconds low speed spin (around 1,000 g), discard flow through
5. Spin 5-10 seconds to eliminate remaining water and transfer the filter units to a sterile centrifuge tube
6. Add 150ul of isolation buffer to each filter unit
7. Using blue pestle and motorized handle, gently macerate the psyllid in the media to expose hemolymph without excessive cell disruption.
8. Spin for 3 minutes at max speed 14000rpm. If recovered volume is less than 150ul, repeat maceration and spin.
9. Discard filter.
10. Resuspend pellet by pipetting.
11. If using multiple batches of psyllid for isolation, pool samples.
12. Gently vortex.
13. Remove a 5ul sample of isolate for initial value.
14. Separate isolate into aliquots for untreated control (water), positive control (triton-x 100 @ final concentration of 0.1%), and desired number of treatments.
15. Add treatments/controls solution at desired concentration. 10x treatment solutions diluted to 1x in isolate are typical.

Incubation and PMAxx Treatment:

16. Aliquot 5ul replicates of each treatment/control into 8 tube strips.
17. Incubate tubes @25ºC on the benchtop
18. Immediately after incubation treat each replicate tube with either 1uL PMAxx or 1 with 1uL water. Incubate out of direct light or covered for 5 minutes and then expose for 15 minutes on a Glo-plate blue LED illuminator or similar cooled 465 nm blue LED light source.

    PMAxx added to 25 uM (1ul of a 5x 0.15mM stock is added to each 5ul replicate)

Light exposure may also be done on a PMAlite in 1.5ml Eppendorf tubes for lower throughput experiments

19. Proceed to DNA extraction with these treated isolations.

    DNA extractions should be done before freezing but samples may then be stored for later PCR

DNA extraction:

20. Add 44uL of lysis buffer to each isolation
21. Under fume hood add 50uL of phenol pH 7~8 to each isolation
22. Vortex briefly to mix, centrifuge at max rpm for 10 minutes
23. Transfer supernatant to new 8-tube strips being careful to not carry forward any phenol
24. Add 25uL of 7.5M ammonium acetate
25. Add 150uL of ice cold 95% EtOH
26. Invert gently and incubate at RT for 10 minutes
27. Centrifuge at max rpms for 10 minutes
28. Discard supernatant without disturbing the pellet
29. Invert tube and allow to air dry
30. Suspend pellet in 50 µL of nuclease-free water
31. Gently vortex or tap/spin to dissolve pellet
32. Store at -20°C until needed for qPCR
